# Substrate recognition and proton coupling by a bacterial member of solute carrier family 17

**DOI:** 10.1016/j.jbc.2023.104646

**Published:** 2023-03-23

**Authors:** Samir Batarni, Nanda Nayak, Audrey Chang, Fei Li, Surabhi Hareendranath, Lexi Zhou, Hongfei Xu, Robert Stroud, Jacob Eriksen, Robert H. Edwards

**Affiliations:** 1 Departments of Neurology and Physiology, UCSF School of Medicine, San Francisco, California; 2 Department of Biochemistry & Biophysics, UCSF School of Medicine, San Francisco, California

**Keywords:** bacterial galactonate transport, proton coupling, solute carrier family (SLC) 17, vesicular glutamate transport

## Abstract

The solute carrier 17 family transports diverse organic anions using two distinct modes of coupling to a source of energy. Transporters that package glutamate and nucleotide into secretory vesicles for regulated release by exocytosis are driven by membrane potential but subject to allosteric regulation by H^+^ and Cl^−^. Other solute carrier 17 members including the lysosomal sialic acid exporter couple the flux of organic anion to cotransport of H^+^. To begin to understand how similar proteins can perform such different functions, we have studied *Escherichia coli* DgoT, a H^+^/galactonate cotransporter. A recent structure of DgoT showed many residues contacting D-galactonate, and we now find that they do not tolerate even conservative substitutions. In contrast, the closely related lysosomal H^+^/sialic acid cotransporter Sialin tolerates similar mutations, consistent with its recognition of diverse substrates with relatively low affinity. We also find that despite coupling to H^+^, DgoT transports more rapidly but with lower apparent affinity at high pH. Indeed, membrane potential can drive uptake, indicating electrogenic transport and suggesting a H^+^:galactonate stoichiometry >1. Located in a polar pocket of the N-terminal helical bundle, Asp46 and Glu133 are each required for net flux by DgoT, but the E133Q mutant exhibits robust exchange activity and rescues exchange by D46N, suggesting that these two residues operate in series to translocate protons. E133Q also shifts the pH sensitivity of exchange by DgoT, supporting a central role for the highly conserved TM4 glutamate in H^+^ coupling by DgoT.

The solute carrier 17 (SLC17) family includes organic anion transporters with diverse physiological roles from excitatory neurotransmission to the recycling of sugars from the lysosome. To subserve these functions, SLC17 family members use two distinct mechanisms. Originally identified as Na^+^-dependent phosphate (Pi) transporters operating at the plasma membrane (1), the founding members were subsequently reported to exhibit a Cl^−^ conductance inhibited by multiple organic anions including urate (2, 3). Subsequent work has shown that these transporters use membrane potential (ΔΨ) to drive the excretion of anionic metabolites and drugs and require activation by Cl^−^ (4, 5, 6). Similarly, the vesicular glutamate transporters (VGLUTs) use ΔΨ as the driving force to package the principal excitatory neurotransmitter glutamate into synaptic vesicles for regulated release by exocytosis (7). They also exhibit a Cl^−^ conductance and allosteric activation by Cl^−^ (8, 9, 10, 11, 12, 13).

In contrast to the dependence on ΔΨ for driving force, the SLC17 member Sialin catalyzes H^+^ cotransport. The electroneutral transport of sialic acid out of lysosomes is driven by the outwardly directed H^+^ gradient (14, 15, 16), recycling the sialic acid derived from lysosomal degradation. Coupling to H^+^ cotransport thus drives anion flux in an opposite direction (from the lysosome into the cytoplasm) from other SLC17 members such as the VGLUTs that use ΔΨ to drive uptake from the cytoplasm into synaptic vesicles. A role for pH in mechanism or regulation has remained unclear for SLC17 family members other than Sialin. However, we recently described allosteric activation of the VGLUTs by lumenal H^+^. This requirement for low pH restricts glutamate uptake to acidic membranes such as synaptic vesicles and prevents the nonvesicular efflux of glutamate by transporter at the cell surface after exocytosis and before endocytosis (12). Thus, closely related SLC17 transporters exhibit two disparate activities both influenced by pH—sialic acid export from lysosomes driven by cotransport of H^+^ and ΔΨ-driven glutamate uptake into synaptic vesicles that is allosterically regulated by lumenal pH. This has raised the possibility that the allostery evolved from an ancestral role for H^+^ in flux coupling.

To understand the role of H^+^ in transport by the SLC17 family, we have studied the *Escherichia coli* D-galactonate transporter DgoT. The *DgoT* gene resides in an operon dedicated to the metabolism and transport of galactonate (17), and in previous work, we showed that DgoT catalyzes the uptake of galactonate by *E. coli* (18). DgoT exhibits remarkable substrate specificity, failing to recognize even gluconate, an epimer of galactonate. We found that DgoT uses H^+^ cotransport to drive galactonate uptake, thus resembling Sialin rather than the VGLUTs. The binding of H^+^ and galactonate presumably drives translocation of the loaded carrier followed by substrate discharge and reorientation of the empty carrier to the periplasm. Membrane potential nonetheless promotes H^+^ flux driven by galactonate, indicating net charge movement not observed for Sialin. We also obtained two crystal structures of DgoT, one oriented to the cytoplasm without substrate and another occluded with substrate facing the periplasm (18). The structures identify the two six-transmembrane-domain (TM) helical bundles characteristic of transporters in the major facilitator superfamily. The structure with substrate shows extensive interactions of DgoT with essentially all recognizable features of galactonate, consistent with the high specificity. In addition, the structures identify a polar pocket within the N-terminal helical bundle (N-domain) that may confer the coupling between substrate and H^+^, and many of the residues implicated show conservation to other members of the SLC17 family including the VGLUTs. To understand the relationship between H^+^ cotransport (by DgoT and Sialin) and allosteric regulation of the VGLUTs by H^+^, we have used a variety of functional assays to test the predictions of the DgoT structures.

## Results

### Substrate recognition by DgoT and Sialin

**Figure 1:**
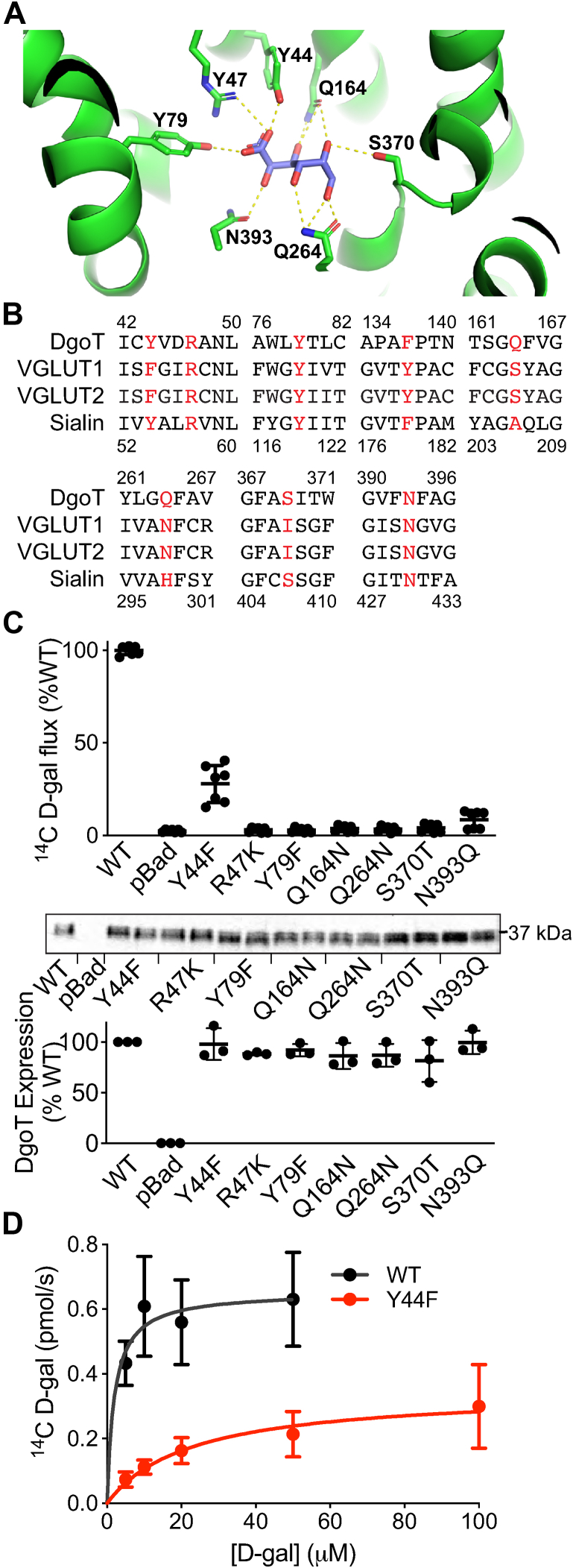
**The stringent requirements of DgoT for substrate recognition.***A*, substrate-binding site in DgoT from DgoT E133Q crystal structure (Protein Data Bank: 6E9O) (18). *B*, sequences of the substrate-interacting residues from DgoT aligned to the homologous sequences from human VGLUT1, 2 and Sialin. The numbers denote the position of residues from DgoT (above) and Sialin (below). *C*, DgoT-deficient *E. coli* strain dgoT727(del)::kan ww transformed with WT, Y44F, R47K, Y79F, Q164N, Q264N, S370T, N393Q DgoT and empty pBad vector. Whole cell uptake of ^14^C-D-galactonate (D-gal) was measured in 150 mM KCl, pH 6 for 2 min at 30 °C. Among the mutants, only Y44F DgoT shows activity above background (*n* = 3). Western blot (below) using a polyhistidine antibody shows equivalent expression of the DgoT constructs. *D*, concentration dependence of ^14^C-D-gal uptake by cells expressing WT and Y44F DgoT, measured in the linear phase of uptake (20 s for WT, 30 s for Y44F). Fitting to the Michaelis–Menten equation was used to determine the K_m_ and V_max_ for WT (1.97 ± 1.1 μM and 0.65 ± 0.05 pmol/s) and Y44F DgoT (22.8 ± 7.7 μM and 0.35 ± 0.04 pmol/s) (*n* = 4).

The outwardly oriented, occluded structure of DgoT shows many interactions with D-galactonate (Fig. 1*A*) (18), consistent with the observed high substrate specificity. A residue conserved throughout almost all SLC17 proteins, Arg47 in TM1 forms a charge pair with the carboxyl group of galactonate. The highly conserved Tyr79 and less conserved Tyr44 also coordinate the galactonate carboxyl (Fig. 1, *A* and *B*). In addition, Gln164, Gln264, Ser370, and Asn393 form a total of eight hydrogen bonds with substrate hydroxyl groups (Fig. 1, *A* and *B*). To determine whether these residues contribute to substrate recognition by DgoT, we replaced them with conservative substitutions that might be expected to preserve folding and activity, and we expressed the mutants in *E. coli* lacking endogenous DgoT. Figure 1*C* shows that almost all of the mutations (R47K, Y79D, Q164N, Q264N, S370T, and N393Q) eliminate D-galactonate uptake by DgoT in intact bacteria where endogenous substrates drive production of a H^+^ electrochemical gradient by the respiratory chain. Western analysis of the bacterial extracts nonetheless shows equivalent expression of wildtype and mutant DgoT (Fig. 1*C*). The lack of activity might not be surprising for mutations involving residues conserved throughout the SLC17 family (Arg47, Tyr79, Asn393) (Fig. 1*B*). However, Gln164 and Ser370 are not conserved and conservative substitutions nonetheless eliminate galactonate transport. Furthermore, replacement of Gln264 by asparagine is not tolerated, even though asparagine occurs at this position in the VGLUTs. Among these mutations, only Y44F preserves activity, and the VGLUTs contain a phenylalanine at this position. In the structure (18), Y44 hydrogen bonds with the carboxyl group of galactonate, and even though the carboxyl of glutamate is positioned similarly in VGLUT2 (19), the interaction must differ, perhaps contributing to the lower apparent affinity for substrate in VGLUT compared with DgoT. Taking advantage of the residual activity, kinetic analysis shows that Y44F reduces both the maximal rate of transport by ∼50% and the apparent affinity, with an increase in K_*m*_ from ∼2 μM to ∼20 μM (Fig. 1*D*). Y44F thus seems to impair substrate recognition, as predicted by its coordination of the D-galactonate carboxyl in the structure (18). However, the residual activity of Y44F also shows that the glutamate carboxyl can be accommodated by a phenylalanine, which occurs at this position in the VGLUTs. We presume that the other mutations also impair substrate recognition, but the absence of activity makes this difficult to test.

**Figure 2:**
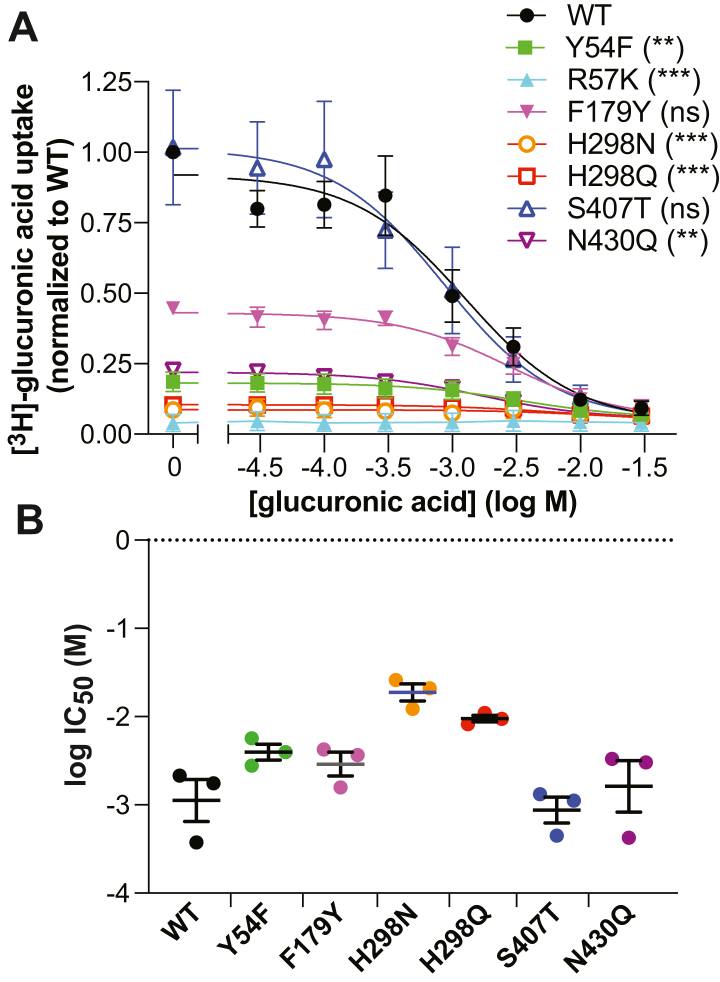
**Effect of mutagenesis on substrate recognition by Sialin.** HEK293T cells expressing internalization-defective Sialin with mutations in the substrate-binding pocket were assayed for uptake of ^3^H-glucuronic acid. Inhibition of uptake by increasing unlabeled glucuronic acid (*A*) with IC_50_ (*B*). ∗∗*p* < 0.01; ∗∗∗*p* < 0.001 by one-way ANOVA for the difference in amount of uptake from WT DgoT (*A*) (n = 3).

In contrast to DgoT, the SLC17 lysosomal sialic acid transporter (Sialin) has relatively low apparent affinity for substrate, with K_*m*_ ∼1 mM (15, 16). It also recognizes multiple organic anions, including glucuronic as well as sialic acid. Although DgoT does not tolerate even conservative substitutions in the residues contacting substrate, the low apparent affinity and lack of substrate specificity suggested that Sialin might differ from DgoT in substrate recognition. Thus, we also made conservative mutations in homologous residues lining the substrate recognition pocket of Sialin (Y54F, R57K, F179Y, H298N/Q, S407T and N430Q) and tested the effect on uptake of ^3^H-glucuronic acid by HEK293T cells transfected with an internalization-defective version of rat Sialin (16). The R57K mutation in TM1 eliminates activity (Fig. 2*A*), consistent with the high conservation of this residue in SLC17, its role in recognition of the substrate carboxyl predicted from the structure of DgoT (18), and the importance of this residue in the VGLUTs (13) (Fig. 1, *A* and *B*). The impact of the conservative lysine substitution indicates that the position of this side chain is as essential as the charge. H298N and H298Q mutations, mimicking residues present in other SLC17 proteins including Gln264 in DgoT and an asparagine in the VGLUTs (Fig. 1, *A* and *B*), also greatly impair glucuronate uptake, but the residual activity exceeds the background in R57K and shows some inhibition by glucuronate (Fig. 2*A*). Similar to mutation of Arg57, Y54F impairs transport by DgoT but exhibits residual activity distinctly above background with an IC_50_ ∼3 mM glucuronate, similar to wildtype Sialin with an IC_50_ ∼2 mM (Fig. 2, *A* and *B*). Coordination of the substrate carboxyl by Tyr54 (as well as Arg57) predicted from the structure of DgoT thus facilitates uptake without substantial contribution to affinity. Replacement of Tyr179 by phenylalanine present in the VGLUTs modestly impairs uptake but without substantial effect on affinity for glucuronate (Fig. 2, *A* and *B*). Although present in the substrate recognition site of DgoT, the homologous residue Phe137 does not appear to interact directly with D-galactonate (18). In striking contrast to the complete loss of function produced by S370T in DgoT, the S407T mutation in Sialin does not impair uptake or recognition of glucuronic acid. The N430Q mutation greatly reduces activity but again without affecting the IC_50_ for glucuronic acid (Fig. 2, *A* and *B*). Thus, Sialin exhibits much less stringent requirements for substrate recognition than DgoT. Although multiple mutations in the binding site impair the rate of transport by Sialin, the affinity for substrate appears relatively unaffected, perhaps consistent with the high K_*m*_ and the recognition of multiple substrates.

### Dependence of DgoT on ΔpH and ΔΨ

**Figure 3:**
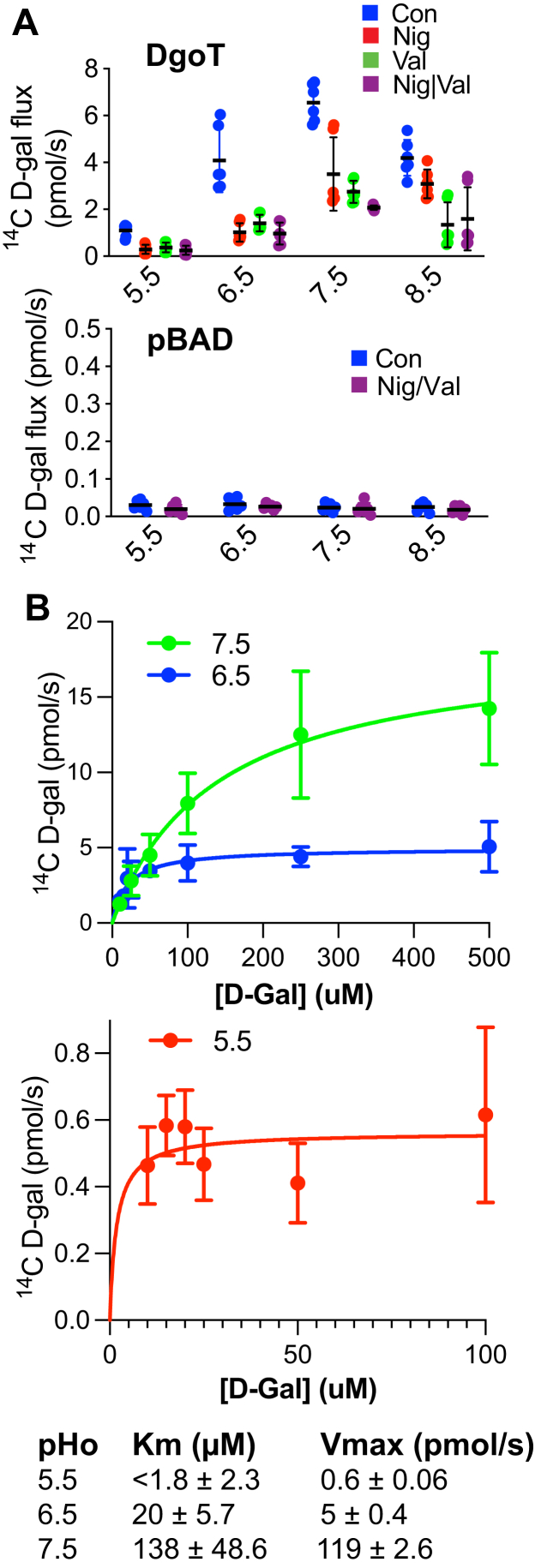
**Uptake by WT DgoT depends on ΔpH and ΔΨ.***A*, uptake of ^14^C-D-gal by spheroplasts from *E*. *coli* expressing WT DgoT and pBad (empty vector) at pH 5.5, 6.5, 7.5, and 8.5, monitored at 2 min in 150 mM K acetate (inside and out), with or without 2 μM nigericin, valinomycin, or both; *n* = 3 for each. *B*, concentration dependence of ^14^C-D-gal uptake by cells expressing WT DgoT monitored at 15 s in pH 5.5, 6.5, or 7.5. The Michaelis–Menten equation was used to fit the data and calculate the K_*m*_ and V_*max*_ at pH 5.5 (1.82 ± 2.3 μM and 0.56 ± 0.06 pmol/s, *n* = 6), 6.5 (20.1 ± 5.7 μM and 4.97 ± 0.41 pmol/s, *n* = 6), and 7.5 (138 ± 48.5 μM and 18.6 ± 2.6 pmol/s, *n* = 3). The Km for pH 5.5 represents an upper bound because the specific activity of the ^14^C-D-gal prevented the use of lower galactonate concentrations.

Previous work has shown that DgoT couples the uptake of D-galactonate to H^+^ symport and transport is electrogenic (18). To determine the relative roles of ΔpH and ΔΨ in the activity of DgoT, we measured uptake into spheroplasts from DgoT^−^ cells transformed with wildtype DgoT or empty vector. Spheroplasts are live bacteria without a cell wall that are still capable of generating a proton electrochemical but, unlike intact bacteria, are sensitive to the K^+^/H^+^ ionophore nigericin and the K^+^ ionophore valinomycin. With symmetric 150 mM K^+^ and the spheroplasts prepared in pH 7, nigericin dissipates ΔpH and valinomycin ΔΨ. Despite the coupling to H^+^ symport, DgoT transports more rapidly at high (6.5–8.5) than low external pH (pH_o_) (5.5) (Fig. 3*A*), with no uptake observed in spheroplasts made from control cells. At low pH_o_ (5.5, 6.5), galactonate uptake depends strongly on both ΔpH and ΔΨ (Fig. 3*A*). Consistent with minimal ΔpH at high pH_o_, nigericin has little effect at external pH 8.5 and progressively larger effect at lower pH_o_ (20). On the other hand, valinomycin shows strong inhibition at all pH_o_, even though ΔΨ is larger at high pH_o_. DgoT thus appears very sensitive to ΔΨ, suggesting a role beyond driving force.

We monitored kinetics directly by varying D-galactonate concentration at different pH_o_. Figure 3*B* shows that V_*max*_ indeed increases more than 100-fold at high pH_o_ and K_*m*_ also increases almost 100-fold from external pH 5.5 (1.8 ± 2.3 μM) to 7.5 (138 ± 48.6 μM). Despite coupling to H^+^, DgoT thus has a higher catalytic activity at neutral than low pH_o_.

**Figure 4:**
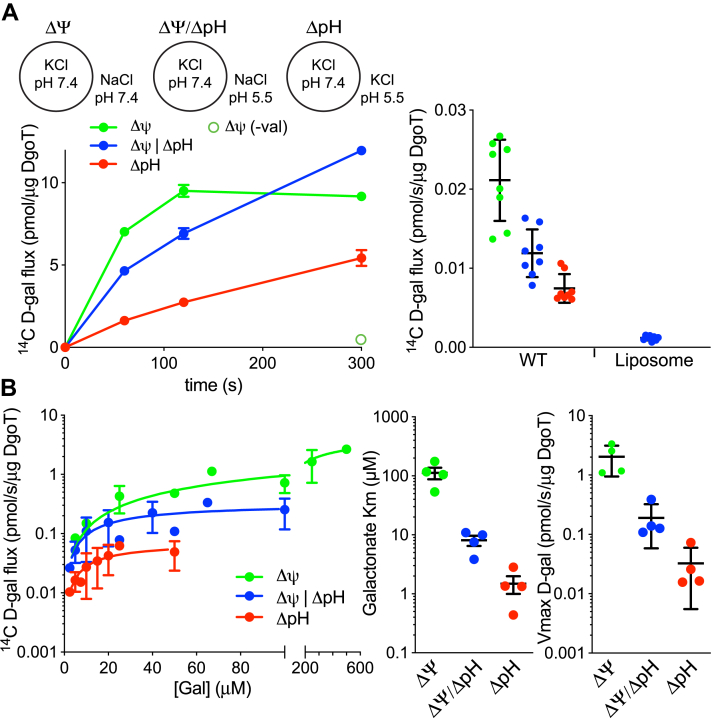
**Uptake by purified, reconstituted DgoT requires both ΔpH and ΔΨ.***A*, purified, WT DgoT was reconstituted into proteoliposomes containing 150 mM KCl, pH 7.4 and uptake of ^14^C-D-gal measured under three different conditions (*left*): membrane potential (ΔΨ) alone, with external 150 mM NaCl, pH 7.4; membrane potential and pH gradient (ΔΨ/ΔpH), with external 150 mM NaCl, pH 5.5; and pH gradient (ΔpH) alone, with external 150 mM KCl, pH 5.5. All conditions were supplemented with valinomycin (200 nM) except for a parallel ΔΨ condition without valinomycin measured only at 5 min (*open green circle*). *Right*, comparison of uptake at 5 min under the three different conditions (*n* = 4). *B*, *left*, concentration dependence of ^14^C-D-gal uptake for 5 min by reconstituted WT DgoT under the three conditions described in (*A*) and supplemented with valinomycin. Using the linear phase of uptake, the Michaelis–Menten equation was used to calculate the K_*m*_ and V_*max*_ of 371 ± 136 μM and 4.49 ± 1.02 pmol/(s x μg) for ΔΨ alone, 24.4 ± 12.2 μM and 0.32 ± 0.07 pmol/(s x μg) for ΔΨ/ΔpH, and 15.63 ± 8.26 μM and 0.072 ± 0.02 pmol/(s x μg) for ΔpH alone (*n* = 4). *Right*, scatter plots comparing K_m_ and V_max_ across three conditions.

Since reliance on the respiratory chain to generate the proton electrochemical gradient Δμ_H+_ makes it difficult to disentangle the independent roles of ΔpH and ΔΨ, we also reconstituted purified DgoT into artificial membranes, using a K^+^ gradient with valinomycin to produce ΔΨ, pH_o_ to create ΔpH, or both. Figure 4*A* shows robust activity above empty liposomes under all three conditions, although uptake is greater when driven by ΔΨ alone, consistent with the faster transport observed at high pH in spheroplasts. The time course also shows that, despite slowing uptake, ΔpH apparently produces higher steady-state concentrations. Kinetic analysis shows a higher K_*m*_ and V_*max*_ when transport is driven by ΔΨ and the lowest with ΔpH alone, with intermediate values in the presence of both chemical and electrical gradients (Fig. 4*B*). Functional reconstitution thus confirms the inverse relationship between speed and apparent affinity, supporting a higher turnover rate in the presence of ΔΨ and neutral pH.

### Proton translocation by DgoT

**Figure 5:**
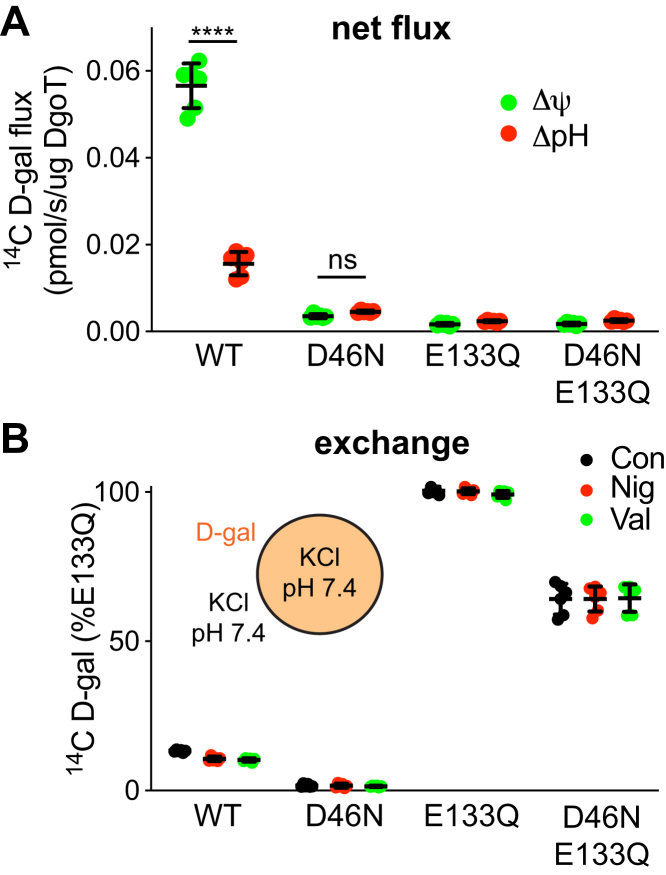
**Role of titratable residues in net flux and exchange by DgoT.***A*, net uptake of ^14^C-D-gal at 5 min by proteoliposomes reconstituted with purified WT, D46N, E133Q, or D46N/E133Q DgoT under the conditions described in legend to Figure 3 to produce either ΔpH or ΔΨ. E133Q reduces net flux to a greater extent than D46N (above) (*n* = 3). D47 and E133 both contribute to the preferential dependence of WT DgoT on ΔΨ (below). *B*, exchange of ^14^C-D-gal for lumenal D-gal for 5 min by WT and mutant DgoT, normalized to E133Q. At pH 7.4 inside and out, nigericin or valinomycin (400 nM each) have no effect, excluding a role for active transport driven by these forces. WT exhibits little exchange and the D46N mutation further impairs this activity, whereas E133Q exhibits robust exchange and rescues D46N (*n* = 3). ∗∗∗∗p < 0.0001; ns, not significant.

Previous work has indicated the importance of both Asp46 and Glu133 for DgoT activity (18) but their role has remained unclear. As Asp46 and Glu133 are titratable residues that can interact with others involved in substrate recognition (*e.g.*, Arg47) (18), their protonation may promote interaction with substrate. To test this possibility, we reconstituted the purified wildtype and mutant proteins into liposomes and used either a pH gradient or membrane potential to drive uptake. As previously demonstrated (18), the D46N mutation greatly reduces uptake, but the residual activity driven by either ΔΨ or ΔpH exceeds the background defined by E133Q and D46N/E133Q mutants (*p* < 0.0001 by *t* test) (Fig. 5*A*). We also find that the residual activity of D46N DgoT differs from wildtype in its dependence on ΔpH relative to ΔΨ. In contrast to the increased transport by DgoT driven by ΔΨ than by ΔpH, D46N in particular shows no difference in uptake between ΔpH and ΔΨ conditions (Fig. 5*A*). What might account for this difference? If neutralization of Asp46 eliminates a site of protonation, this would alter the stoichiometry of H^+^ coupling, rendering DgoT less dependent on ΔΨ.

To test the role of Asp46 and Glu133 in H^+^ coupling, we first took advantage of the reconstitution system and monitored exchange of external, radiolabeled galactonate for internal unlabeled galactonate under conditions of symmetric pH with no membrane potential. Figure 5*B* shows that wildtype DgoT confers low but detectable levels of exchange, and this does not reflect net uptake because it is not inhibited by nigericin or valinomycin. The D46N mutation impairs exchange, but E133Q greatly increases exchange relative to wildtype and again the ionophores have no effect, excluding net flux. Since these mutations mimic protonation, we infer that protonation of Glu133 promotes substrate recognition and enables movement of the loaded carrier. Glu133 in DgoT thus plays a central role in coupling protonation to substrate translocation. In contrast to reversible protonation of the wildtype protein, the irreversible nature of these mutations prevents the loss of H^+^ required for movement of the unloaded carrier and net flux, very similar to Glu325 in lac permease (21). Importantly, the E133Q mutation rescues exchange by D46N, suggesting that Asp46 may relay protons to Glu133 and the E133Q mutation bypasses the defect in this relay.

**Figure 6:**
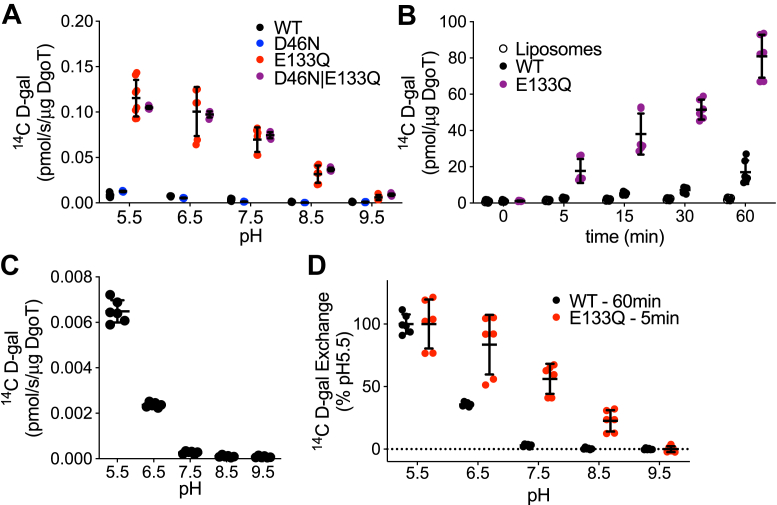
**Effect of mutations on the pH dependence of exchange by reconstituted DgoT.***A*, exchange of ^14^C-D-gal for D-gal by WT and mutant DgoT at pH 5.5, 6.5, 7.5, 8.5, and 9.5. The D46N mutant resembles WT DgoT in pH dependence of exchange and E133Q/D46N the single mutant E133Q (n = 6–8). *B*, time course of exchange by empty liposomes, WT, and E133Q DgoT at pH 5.5 shows that WT DgoT exhibits exchange not observed for empty liposomes but functions more slowly (*n* = 6). *C*, pH dependence of exchange for 1 h by proteoliposomes reconstituted with WT DgoT (*n* = 6). *D*, pH dependence of exchange by WT DgoT for 1 h and E133Q for 5 min, normalized to each at pH 5.5. The E133Q mutation shifts the activation of exchange to higher pH (n = 6).

Exchange mediated by wildtype DgoT presumably requires protonation of Glu133. To test this possibility, we monitored exchange activity at different pH, but exchange by wildtype DgoT is much less active than that of E133Q and the D46N/E133Q mutants even at the lowest pH tested (5.5) (Fig. 6*A*). To compare exchange by wildtype and E133Q DgoT, we prolonged the reaction, observing much more robust exchange by wildtype at 1 h, particularly at low pH_o_ (Fig. 6, *B* and *C*). Indeed, wildtype DgoT exhibits a relatively steep dependence on low pH_o_ (at 6.5 and especially 5.5), which is missing from the E133Q mutant. This suggests that Glu133 confers the dependence of wildtype DgoT on low pH. However, exchange by E133Q DgoT still shows progressive activation over a wide range of pH, suggesting additional sites of protonation with a range of pKa (Fig. 6, *A* and *D*). Again, the D46N/E133Q double mutant behaves similarly to E133Q, supporting a primary role for Glu133.

**Figure 7:**
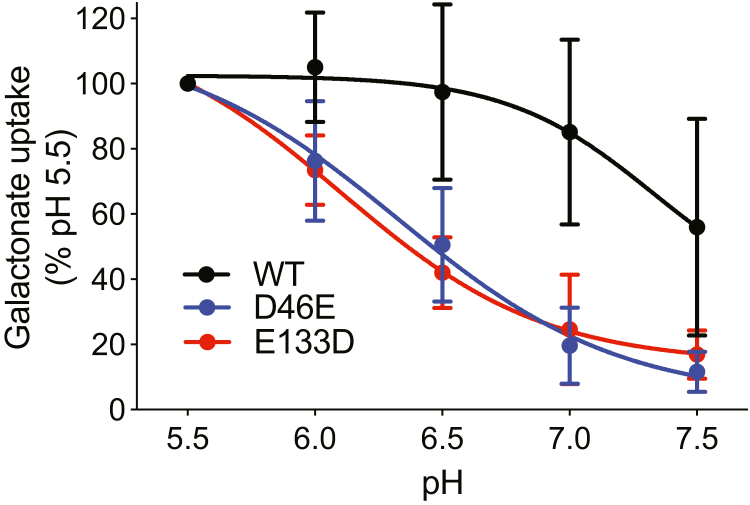
**Conservative substitutions at D46 and E133 alter the pH sensitivity of DgoT.** DgoT-deficient *E. coli* were transformed with WT, D46E, E133D, D46E/E133D DgoT and empty pBad vector, and ^14^C-D-gal uptake by whole cells was measured for 5 min as given in the legend to Figure 1. All the mutations shift the activation of DgoT to lower pH, but the effect of D46E is restricted to pH > 6 (n = 4).

To test further the roles of Asp46 and Glu133 in H^+^ translocation, we made conservative mutations at each position and determined the effect on pH dependence of net flux by intact DgoT^−^ bacteria transformed with wildtype, D46E, or E133D DgoT. In contrast to spheroplasts and proteoliposomes, intact bacteria show increased activation of galactonate uptake by pH < 7.5 (Fig. 7), presumably due to differences in regulation of Δμ_H+_. Both conservative mutations affect this pH sensitivity, requiring lower pH for maximal activation and hence indicating reduced pH sensitivity. The length of the side chain at these positions is thus tuned for optimal pH sensitivity, presumably due to the effect of local environment on their pKa since the intrinsic pKa of aspartate (3.9 at 25 °C) is lower than that of glutamate (4.07 at 25 °C). The analysis of net flux thus supports the role of Asp46 and Glu133 in pH sensitivity of DgoT.

## Discussion

The results indicate different modes of substrate recognition by bacterial DgoT and mammalian Sialin. Consistent with its high specificity for D-galactonate, DgoT does not tolerate even conservative mutations at most of the residues that interact with substrate. The sensitivity to mutation suggests extremely precise requirements for the occluded state with substrate bound, and that is presumably how DgoT can distinguish between galactonate and such closely related compounds as its epimer gluconate (18). In contrast, Sialin shows much less sensitivity to conservative mutation at the homologous residues. Several of the mutations impair but do not eliminate transport by Sialin, and the sensitivity to inhibition by glucuronate does not change significantly for most of these. This suggests a distributed mechanism for substrate recognition by Sialin and an effect of multiple mutations on kinetics rather than affinity, consistent with the high K_*m*_ of Sialin and its recognition of multiple substrates from glucuronic to sialic acid.

The structure of DgoT enables us to interpret several of the differences in sensitivity to mutation of the two transporters. In particular, S407T in Sialin does not impair glucuronate uptake but the homologous mutation in DgoT (S370T) eliminates galactonate transport. In the structure of DgoT, Ser370 interacts with the C4 hydroxyl of galactonate (18) and the ability to distinguish between epimers may depend on serine rather than threonine.

Although DgoT is in general more sensitive to mutation than Sialin, several residues appear critical for both transporters. Replacement of the TM1 arginine (Arg47 in DgoT, Arg57 in Sialin) by lysine effectively eliminates transport by both proteins, consistent with the high conservation of this residue in SLC17 and its interaction with the substrate carboxyl in DgoT (18). Like Q264N in DgoT, mutation of His298 at the same position in Sialin to either glutamine (as in DgoT) or asparagine (as in the VGLUTs) greatly impairs transport activity. Gln264 presumably does not tolerate replacement by asparagine because glutamine contacts the C6 hydroxyl group of D-galactonate (18) and the shorter side chain of asparagine prevents this interaction. But why is glutamine at this position tolerated in DgoT but not Sialin? Galactonate and glucuronate differ in substituent at the C6 position, with a hydroxyl in galactonate and aldehyde in glucuronate. However, glucuronate exists primarily in the ring form, similar to sialic acid, whereas galactonate is strictly linear and this may contribute to the requirement for histidine in Sialin. The N393Q mutation in DgoT and N430Q mutation at the same position in Sialin both severely impair transport, and Asn393 coordinates the C2 and C4 hydroxyls of galactonate (18), suggesting that the length of this side chain may be important to accommodate the different epimers.

Surprisingly, a TM1 tyrosine shows more sensitivity to mutation in Sialin than DgoT. The Y44F mutant in DgoT confers some residual transport activity and the K_*m*_ is 10-fold higher than wildtype DgoT, confirming the importance of this residue predicted from the structure to coordinate the substrate carboxyl (18). However, the Y54F mutation at the same position in Sialin almost eliminates glucuronate transport, even though galactonate and glucuronate do not differ at the C1 carboxyl. Tyr54 presumably plays a role beyond substrate recognition in the transport mechanism.

We also find that, despite the mechanism of H^+^ symport, DgoT depends strongly on ΔΨ. Spheroplasts show faster transport at high pH_o_, where ΔΨ predominates, similar to bacterial lactose transport (22). Proteoliposomes reconstituted with DgoT also show more activity when driven by ΔΨ than by ΔpH. On the other hand, ΔpH confers uptake with lower K_*m*_, suggesting increased substrate affinity and this or a role for ΔΨ in a rate-limiting step of the transport cycle that may contribute to the reduced V_*max*_ and lower K_*m*_. These observations indicate electrogenic transport and suggest a H^+^:galactonate stoichiometry >1:1. Transport into intact bacteria shows a somewhat different pH dependence with maximal rates at low pH, and the difference from proteoliposomes with recombinant protein and defined ionic conditions suggests a role for regulated expression of bacterial Δμ_H+_ as ΔpH and ΔΨ rather than any intrinsic differences in the properties of DgoT.

The parallel increase in both K_*m*_ and V_*max*_ with galactonate transport driven by ΔΨ might seem to reflect the relationship K_*m*_ = (K_*off*_ + K_*T*_)/K_*on*_, where K_*off*_ is the dissociation rate for galactonate, K_*T*_ the rate of translocation, and K_*on*_ the association rate. An increase in K_*T*_ due to transport driven by ΔΨ rather than ΔpH could increase K_*m*_ without changing K_D_ (K_*off*_/K_*on*_). However, not all transporters exhibit a direct correlation between K_*m*_ and V_*max*_. Lac permease resembles DgoT in its primary dependence on ΔΨ rather than ΔpH (23). However, ΔΨ lowers the K_*m*_ of lac permease despite increasing the V_*max*_. Since increased V_*max*_ presumably reflects increased K_*T*_, the lowered K_*m*_ under energized conditions must reflect a reduced K_*D*_ in the case of lac permease. Thus, DgoT appears to differ from lac permease in the effect of ΔΨ on K_*D*_, with no effect in the case of DgoT and a reduction in the case of lac permease. It will be interesting to determine whether the apparent invariance of substrate recognition with changes in ΔΨ is a more general feature of the SLC17 family.

We further identify the residues that mediate coupling to H^+^. The E133Q mutation promotes exchange relative to wildtype DgoT, implicating protonation of this residue in translocation of the carrier with substrate. The E133Q mutant cannot catalyze net flux because movement of the unloaded carrier requires deprotonation of this residue, very similar to the effect of Glu325 mutation in lac permease (21). Consistent with this, the activation of exchange by wildtype DgoT at low pH_o_ shifts to higher pH_o_ in E133Q, indicating the loss of an important, required protonation site (Glu133) with a lower pKa and the persistence of other sites with higher pKa that, with Glu133 neutralized, enable activation of exchange at higher pH_o_. The analysis of conservative substitutions at Asp46 and Glu133 confirms the importance of these residues for pH sensing by DgoT. The mutations shift the requirement for allosteric activation to lower pH, indicating reduced sensitivity to H^+^ and supporting the role of these residues in H^+^ coupling. The similar effect of both conservative mutations, one from glutamate to aspartate and the other from aspartate to glutamate, further indicates the importance of local environment rather than an intrinsic property of the side chain in determining pKa.

The importance of Asp46 and the electrogenic nature of transport by DgoT raise the possibility that, in addition to protonation of Glu133, protonation of Asp46 confers coupling of galactonate flux to H^+^, thereby conferring net charge movement. The D46N mutation greatly impairs but does not eliminate transport, with residual net flux above that observed with E133Q. Membrane potential can still promote the residual transport catalyzed by D46N DgoT, possibly by accelerating a step in the transport cycle without net charge movement coupled to flux. In addition, the E133Q mutation bypasses the requirement for Asp46 in exchange, and the double D46N/E1334Q mutant shows the same pH sensitivity as the E133Q alone. Thus, Asp46 does not have an independent role in H^+^ coupling but rather appears to work with Glu133. The position of Asp46 at the end of a tunnel from the periplasm to a polar pocket within the N-domain and the proximity to Glu133 (18) further suggest that Asp46 may serve to shuttle H^+^ to Glu133. The residual pH sensitivity of E133Q/D46N DgoT indicates either that a different titratable residue mediates direct coupling to H^+^ or that protonation at other sites confers allosteric activation of transport.

What can the H^+^ coupling of DgoT tell us about the allosteric interaction of VGLUTs with H^+^? The two mechanisms may be related, and it is possible that the TM4 glutamate, which is highly conserved across SLC17, controls availability of the TM1 arginine for interaction with the carboxyl of glutamate as well as of galactonate, but without net H^+^ flux. However, the persistent pH sensitivity of exchange by E133Q DgoT raises the possibility that the other, unidentified site predicted to recognize a second H^+^ by DgoT has acquired an allosteric role.

## Experimental procedures

### Chemicals

Sodium D-galactonate was prepared as described (18) from the calcium salt using oxalic acid to chelate the Ca^++^, NaOH to adjust the pH, and ethanol to precipitate the Na galactonate. The resulting Na galactonate crystals were stored at room temperature. The ^14^C-D-galactonate was obtained from American Radiochemicals Corp.

### DgoT mutagenesis, expression, and purification

Full-length *E*. *coli* DgoT with a C-terminal modified decahistidine tag was subcloned into pBAD; mutations were introduced by site-directed mutagenesis and confirmed by sequencing.

*E*. *coli* C41 cells were transformed with WT, D46N, E133Q, and D46N/E133Q DgoT and grown at 37 °C in TB supplemented with 2 mM MgSO_4_ and 100 μg/ml carbenicillin. At *A*_600_ ∼0.6 to 0.8, the culture was induced with 0.1% L-Arabinose (Sigma-Aldrich) followed by incubation overnight at 18 °C. A typical yield of 200 g from 6 L culture was split into 50-g aliquots and stored at −80 °C. Harvested cells (50 g) were resuspended in 20 mM Tris (pH 7.4), 150 mM NaCl (5 ml/1 g cell mass = 250 ml) containing 1 × protease inhibitors (Sigma) and lysed using the Emulsiflex C3 homogenizer (ATA Scientific) for six cycles at 15,000 to 20,000 psi. Debris was sedimented at 20,000*g* for 30 min, and the supernatant sedimented at 185,500*g* for 1 h to collect the membranes. The membranes were split into 3-g aliquots, flash-frozen in liquid nitrogen, and stored at −80 °C until further use.

A 3-g aliquot of membranes was resuspended in 50 ml 20 mM Tris (pH 7.4), 150 mM NaCl, 10% glycerol, 1.4% (w/v) n-dodecyl-β-maltoside (β-DDM; Anatrace) (solubilization buffer) using a glass Dounce homogenizer. The membranes were solubilized by stirring at 4 °C for 2 h, the insoluble material was removed by sedimentation at 185,500*g* for 20 min, and the supernatant was incubated with 5 ml Talon cobalt affinity resin (Clontech) at 4 °C for 1 h, with rocking. The resin was then washed with 10 column volumes of 20 mM Tris (pH 7.4), 150 mM NaCl, 10% glycerol, 0.05% β-DDM, 10 mM imidazole (wash buffer), followed by two column volumes of wash buffer containing 500 mM NaCl, and then by two column volumes of wash buffer. The protein was eluted with four column volumes of wash buffer containing 150 mM imidazole (elution buffer). Imidazole was removed from the eluate with a 10-DG desalting column (Bio-Rad), the protein concentrated to 0.5 ml using a 50 kDa molecular weight cut-off (MWCO) centrifuge concentrator (Millipore) followed by filtration through 0.2-μm PVDF. To switch the detergent from β-DDM to β-NG (Anatrace), 0.5 ml protein was injected onto a Superose 6 column (Sigma-Aldrich) pre-equilibrated with 10 mM Hepes (pH 7.4), 150 mM NaCl, 10% glycerol, 0.5 mM TCEP, and 0.2% β-NG (S buffer). Peak fractions were pooled and stored at −80 °C until further use.

### Whole cell uptake

DgoT-deficient *E*. *coli* strain dgoT727(del)::kan (Keio collection; *E*. *Coli* Genetic Stock Center, Yale) was transformed with WT, D46N, E133Q, D46N/E133Q, D46E, E133D, D46E/E133D, Y44F, R47K, Y79F, Q164N, Q264N, S370T, and N393Q DgoT constructs in pBAD. For each construct, a single colony was grown in 2 ml LB (supplemented with 100 μg/ml carbenicillin and 35 μg/ml kanamycin) overnight at 37 °C and glycerol stocks (25%) were stored at −80 °C. The glycerol stocks were then used to inoculate 4 ml M63 minimal media containing 15 mM (NH_4_)_2_SO_4_, 100 mM KH_2_PO_4_ (pH 7.0), 1.8 μM FeSO_4_⋅7H_2_O, 1 mM MgSO_4_⋅7H_2_O, 0.2% glycerol, 5 × 10^−5^% thiamine, and 0.1% casamino acids (+100 μg/ml carbenicillin and 35 μg/ml kanamycin) and grown overnight at 37 °C. WT, mutant DgoT, and pBad (empty vector) were grown by adding 1.2 ml saturated culture into 40 ml M63 minimal media supplemented with the same antibiotics. Cultures were grown at 37 °C with shaking until *A*_600_ ∼0.6 to 0.8 and induced with 0.1% L-arabinose at 37 °C for 3 h. Cells were harvested at 3500*g* for 10 min at 4 °C, the pellet was washed twice with 25 ml 10 mM Mes (pH 6), 150 mM KCl (MK buffer), and resuspended to *A*_600_ ∼2.0. To measure flux, 50 μl cell suspension was preincubated at 30 °C for 5 min. The reaction was initiated by addition of 450 μl MK buffer containing 10 μM ^14^C-galactonate (also prewarmed to 30 °C) and the mixture incubated at 30 °C for 0 to 5 min. The reaction was terminated with 1 ml cold MK buffer, filtered through 0.45-μm nitrocellulose (Millipore), washed twice with 2 ml cold MK buffer, and the bound radioactivity measured by scintillation counting. For the measurement of kinetics, background uptake extrapolated from the effect of increasing nonradioactive galactonate on uptake of ^14^C-galactonate was subtracted from the raw uptake at each galactonate concentration; the total galactonate accumulated was then calculated from the ratio of nonradioactive/radioactive galactonate. All transport assays were performed in duplicate using at least three independent preparations.

The expression of DgoT was determined by immunoblotting the lysates with a polyhistidine antibody conjugated to horseradish peroxidase (HRP; Qiagen), with chemiluminescent detection using a ChemiDoc MP imaging system (Bio-Rad).

### Spheroplast preparation and uptake

DgoT constructs in pBad were transformed into the *E*. *coli* DgoT knockout strain, and glycerol stocks were used to inoculate 45 ml M63 minimal media (+100 μg/ml carbenicillin and 35 μg/ml kanamycin) for growth overnight at 37 °C. The overnight culture (30 ml) was used to inoculate 1 L M63 minimal media (+carbenicillin/kanamycin), grown at 37 °C to *A*_600_ ∼0.6 to 0.8, and expression induced with 0.1% L-arabinose for either 3 h at 37 °C for kinetic measurements or overnight (16–18 h) at 18 °C for other assays. Cells were harvested at 6000*g* for 10 min, the pellet was washed in 200 ml 30 mM Tris (pH 8, 20 ml per 0.2 g cell pellet), resuspended in 110 ml 30 mM Tris (pH 8) with 20% sucrose (50–60 ml buffer per g cell pellet), and incubated for 15 min at room temperature. Lysozyme was added to a final concentration of 20 μg/ml and the mixture incubated for 10 min at room temperature. After 1:1 dilution with 30 mM Tris (pH 8), EDTA was added to a final concentration of 1 mM, incubated for 15 min with gentle stirring, and 7 U/ml Benzonase (Sigma-Aldrich) added to fragment released DNA. The spheroplasts were then sedimented at 16,000*g* for 20 min and resuspended in 20 mM Hepes (pH 7), 150 mM KCO_2_CH_3_, 20% (w/v) sucrose, and 5 mM MgSO_4_ (supplemented with 20 mM glycerol) to an *A*_600_ ∼7.0.

Spheroplasts and reaction buffers were preincubated at 30 °C for at least 5 min and reactions initiated by addition of 50 μl spheroplast solution to 450 μl reaction buffer containing 20 mM buffer (Mes pH 5.5 and 6.5; Hepes pH 7.5, or TRICINE pH 8.5), 150 mM KCO_2_CH_3_, and 20% (w/v) sucrose, 5 mM MgSO_4_, 10 μM ^14^C-galactonate, with or without either 2 μM nigericin or 2 μM valinomycin or 0 to 500 μM nonradioactive galactonate for the measurement of kinetics. The reactions were incubated either for 2 min or 15 s for the kinetic assays. After incubation, the reactions were terminated with 1 ml cold reaction buffer followed by filtration through 0.45-μm nitrocellulose, two washes with 2 ml cold reaction buffer, and measurement of the bound radioactivity by scintillation counting. For the measurement of kinetics, background uptake extrapolated from the effect of increasing nonradioactive galactonate on uptake of ^14^C-galactonate was subtracted from the raw uptake at each galactonate concentration as for uptake by intact cells. All transport assays were performed in duplicate using at least three independent preparations.

### DgoT reconstitution and radiotracer flux assay

Purified DgoT was reconstituted using preformed liposomes permeabilized with Triton X-100 (24). *E*. *coli* polar lipids (10 mg; Avanti Polar Lipids) were dissolved in 1 ml chloroform and dried under nitrogen while rotating in a round bottom Pyrex glass bulb (to create a thin film capable of forming unilamellar vesicles) and then under vacuum overnight. The dried lipid film was rehydrated in 1 ml 20 mM Hepes (pH 7.4), 150 mM KCl (reconstitution buffer) by incubation at 37 °C for 30 min, and resuspended by pipetting. The suspension was transferred to a 15-ml conical tube, subjected to three freeze–thaw cycles between liquid nitrogen and ambient RT, then transferred back to the round bottom Pyrex glass bulb and sonicated in a water bath until optically clear. The lipids were then extruded by 10 passages through a 400-nm filter at 30 to 35 °C.

To prepare proteoliposomes, the liposomes were destabilized by adding 0.24% (v/v) Triton X-100 (2.4 μl in 1 ml lipid suspension), which produced 80% maximum *A*_540_ in a liposome destabilization curve, and nutated for 30 min at 4 °C. Purified DgoT (or equal volume of buffer for control) was added to the destabilized liposomes at a 1:50 protein:lipid (w:w) ratio and incubated for 15 min at 4 °C. To extract detergent, SM2 Bio-beads (Bio-Rad) were added sequentially in three steps. First, 0.05 g/ml SM2 Bio-beads were added and nutated for 1 h at 4 °C. The addition of Bio-beads was repeated (step 2) and incubated overnight at 4 °C. For the final step, 0.08 g/ml SM2 Bio-beads were added and nutated for 2 h at 4 °C. The Bio-beads were removed by repeated passage through a 26g needle. Proteoliposomes were harvested by sedimentation at 300,000*g* for 30 min and resuspended to a concentration of 2 mg/ml lipids in lumenal reconstitution buffer containing 20 mM Hepes (pH 7.4) and 150 mM KCl (lumenal buffer). Proteoliposomes were flash-frozen in liquid nitrogen and stored at −80 °C until further use.

To measure net flux and kinetics, the proteoliposomes were thawed, extruded by 10 passages through a 400-nm filter at 30 to 35 °C and washed twice with lumenal buffer after sedimentation at 300,000*g* for 15 min and resuspension in external buffer containing, for ΔΨ, 20 mM Hepes (pH 7.4), 150 mM NaCl; for ΔΨ/ΔpH, 20 mM Mes (pH 5.5), 150 mM NaCl; or for ΔpH, 20 mM Mes (pH 5.5), 150 mM KCl. Transport was initiated by adding 50 μl proteoliposomes (2 μg protein) to 450 μl reaction buffer containing the three different conditions (for ΔΨ, ΔΨ/ΔpH, and ΔpH) supplemented with 0.2 to 0.4 μM valinomycin and 5 to 10 μM ^14^C-galactonate. Unless otherwise noted, net flux and exchange were monitored at 0 and 5 min. Kinetics were measured at 30, 60, and 120 s corresponding to the linear phase of uptake for each respective condition.

For exchange, proteoliposomes were thawed, 10 mM galactonate was added, and the mixture was freeze-thawed twice in liquid nitrogen/RT (with gentle mixing at RT), extruded, washed twice, and resuspended in lumenal buffer. Reaction solutions for exchange contained 20 mM buffer (Mes pH 5.5 and 6.5, Hepes pH 7.5, TRICINE pH 8.5, or CAPSO pH 9.5), 150 mM KCl, 10 μM ^14^C-galactonate, 0.4 μM nigericin, and 0.4 μM valinomycin. Both proteoliposomes and reaction buffer were preincubated at 30 °C for at least 5 min before initiating the reactions by adding 20 μl proteoliposomes (0.8 μg protein) to 180 μl reaction buffer. The reactions were terminated with 1 ml cold reaction buffer followed by filtration through 0.45-μm nitrocellulose, washing twice with 2 ml cold buffer, and measurement of the bound radioactivity by scintillation counting. The zero time point was subtracted as background for both net flux and exchange. Kinetic experiments were normalized to controls eliminating ΔΨ or ΔpH or both using nigericin or valinomycin (0.2 μM for 5 min, 0.4 μM for kinetics): no ionophore (for ΔΨ), nigericin (for ΔΨ/ΔpH and ΔpH). All transport assays were performed in duplicate on at least three independent preparations from two independent reconstitutions.

### Sialin transport activity

HEK293T cells were transfected with pCDNA3.1 encoding WT Sialin-HA and the indicated mutants HA-tagged in the first extracellular loop. One day after transfection, the cells were seeded into 24-well plates treated with poly-L-lysine (0.1 mg/ml). The next day, the cells were assayed for ^3^H-glucuronic acid uptake. Cells were washed once in Ringer’s solution before incubation with ^3^H-glucuronic acid and from 0 to 25 mM cold glucuronic acid for 4 min at room temperature. The reaction was stopped by washing the cells twice with cold Ringer’s solution. The cells were then lysed in 1% SDS, and the lysate radioactivity was measured using a scintillation counter. Activity was represented as percent WT.

## Data availability

All of the data are contained within the article.

## Data analysis and statistics

All data were plotted and statistics determined using GraphPad Prism software. In the graphs, the bars indicate mean and error bars SEM.

## Conflict of interest

The authors declare that they have no conflicts of interest with the contents of this article.
